# Ecology, ethology, and evolution in the Anthropocene

**DOI:** 10.1242/bio.060175

**Published:** 2024-03-01

**Authors:** Matheus Sanita Lima, Frederick Curtis Lubbe, Sarah Helen Dias dos Santos, Stefane Saruhashi, Jorden Mikaela Maglov, Joseane Moreira do Nascimento, Soren Zachary Coulson

**Affiliations:** ^1^ Department of Biology, Western University, London, Ontario N6A 5B7, Canada; ^2^ Institute of Botany of the Czech Academy of Sciences, v.v.i, Dukelská 135, 37901, Třeboň, Czech Republic

**Keywords:** Anthropogenic, Climate change, Biodiversity loss, Planetary boundaries, Living planet index, Great acceleration

## Abstract

The 53rd Ontario Ecology, Ethology, and Evolution Colloquium (OE3C 2023) took place at Western University (London, Canada) on 25-27 May 2023, attracting 160 participants. This Meeting Review aims not only to recapitulate what was discussed during the event, but also to provide a brief synthesis of how biologists can move forward. The event was organised and run by graduate students and postdoctoral researchers from the Department of Biology at Western University. With three international keynote speakers, seventy talks, and fifty poster presentations, the OE3C 2023 spanned a wide range of contemporary research in Ecology, Ethology, and Evolution (“the 3 E's”). The colloquium theme was “Surviving the Anthropocene: future steps for the 3 E's under pressing planetary issues”, which was complemented by illustrations depicting the fauna and flora of the “Canadian Anthropocene”. Participants discussed what biologists and researchers can do regarding future climate and environmental catastrophes. The meeting culminated in a panel discussion comprising three climate change specialists who examined topics such as the Anthropocene and the Great acceleration, the living planet index, and carbon bombs. Although not exhaustive, these topics served as a starting point for the necessary discussions about how biologists can contribute to the fight for the survival of life on Earth.

## Teaser

**Summary:** This Meeting Review summarizes the panel discussions during the 53rd Ontario Ecology, Ethology, and Evolution Colloquium (OE3C 2023), which centred on surviving the Anthropocene under pressing planetary issues.

## Introduction

For the past 52 years, the Ontario Ecology, Ethology, and Evolution Colloquium (OE3C) has brought together student and early career ecologists, ethologists, evolutionary biologists and alike to present their work. Each annual edition boasts a different theme that should encapsulate the most pressing issues (or the most promising avenues of research) for the 3 E's. Evolution has always been the tacit common link amongst the colloquium's disciplines, but our choice of the Anthropocene was considered timely.

**Fig. 1.**
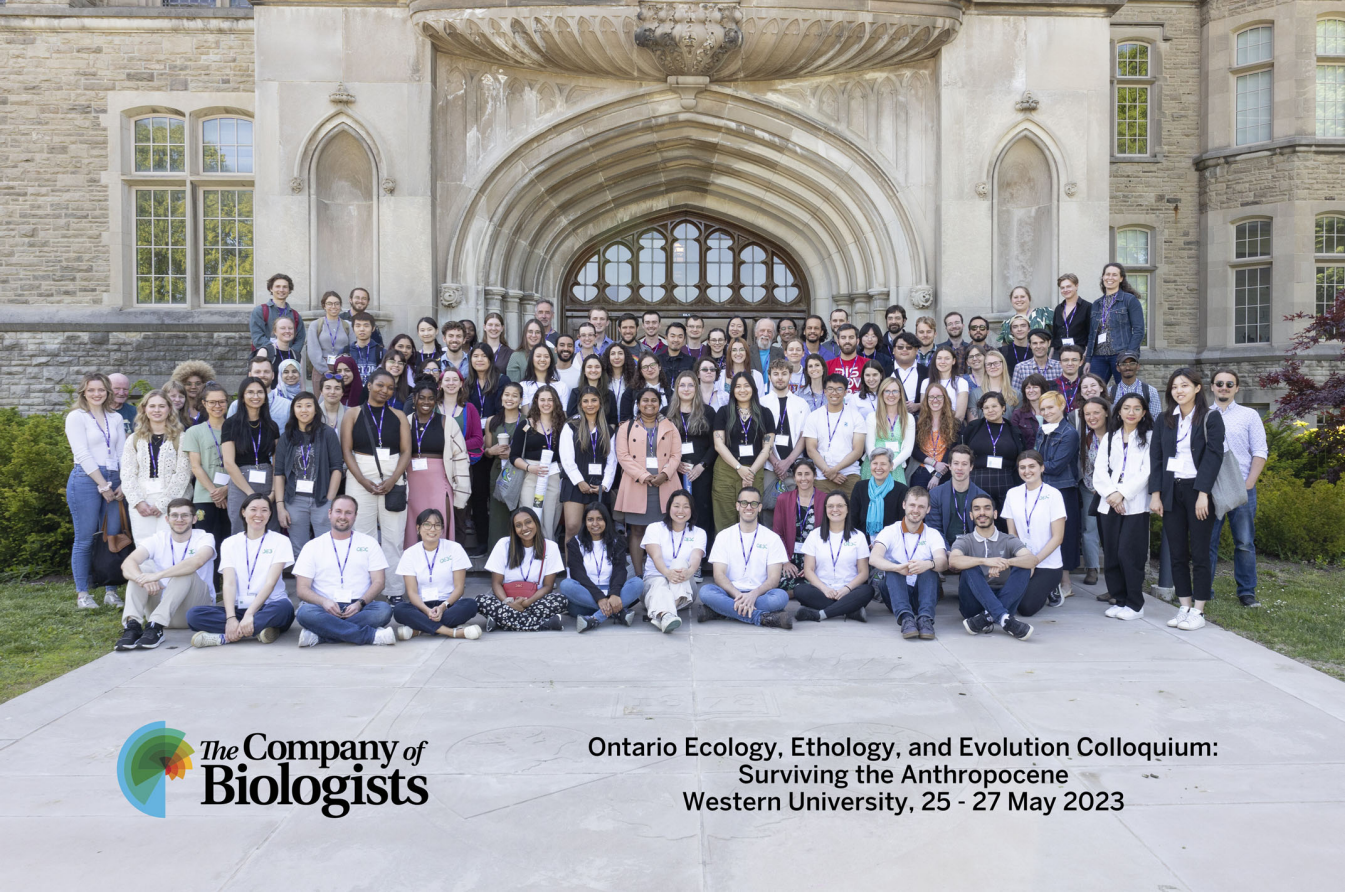
**Group photo taken in front of the University College Building at Western University (London, Canada).** Students (undergraduate and graduate), postdoctoral researchers, and faculty members met on 25–27 May 2023 to present their work on ecology, ethology, and evolution at the OE3C 2023.

“Surviving the Anthropocene: future steps for the 3 E's under pressing planetary issues” was chosen as the overarching theme with the goal to allow both early career and established biologists to discuss what we can do in the face of our current planetary crisis. Biologists from 19 Ontarian universities (Fig. 1) convened and presented research spanning numerous aspects of ecology, ethology, and evolution. In the end, we realised that even projects that were not explicitly concerned with the Anthropocene could not avoid a connection with it. As humans have pervasively shaped planet Earth, biologists now find their study subjects inevitably under the hidden or explicit influence of anthropogenic forces.

The OE3C 2023 roster was composed of 134 students (both undergraduate and graduate), four postdoctoral researchers, and 22 faculty members. The participants exhibited various degrees of familiarity with the Anthropocene, an overwhelming topic even for more experienced researchers. Dealing with a planetary existential crisis created by our own species is inherently daunting, but a sense of agency and an unabated eagerness to learn also transpired during talks, coffee breaks, and networking sessions. Beyond concurrent sessions, on-campus excursions, plenary speakers, and a panel discussion (https://oe3c2023.com/), the OE3C 2023 had mascots that illustrated different “stories of the Anthropocene”. Attendees left the event well informed and ready to continue their journeys through our uncertain times.

This Meeting Review aims not only to recapitulate what was discussed during the event, but also to provide a brief synthesis of how biologists can move forward. Ideally, this paper will serve as a primer or a dispatch to give biologists a starting point into the discussions around the Anthropocene. The use of art to communicate complex (and overwhelming) scientific concepts is highlighted. We believe biologists, regardless of their chosen subfield of study, are strategically positioned to assess, predict, and mitigate what lies ahead of life on Earth.

## The Anthropocene

The term Anthropocene has a long and complex history, with informal use dating to the 1980s with Eugene Stoermer (Steffen et al., 2011) and official and more popular usage in the 2000s (Crutzen and Stoermer, 2000; Crutzen, 2006a). They described the Anthropocene as our current geological epoch, marked by unprecedented ecological disturbance (in a global scale) caused by humans. However, discussions regarding the potential impacts of human activities began far before the term Anthropocene was coined, with ongoing debate since the mid-19th century, and concepts such the “Anthropozoic Era”, the “Noösphere”, the “Anthrocene”, the “Capitalocene”, the “Homogenocene”, the “Anthroposphere”, and “Anthromes” have all been proposed (Samways, 1999; Ellis et al., 2010; Steffen et al., 2011; Baccini and Brunner, 2012; Lewis and Maslin, 2015; Moore, 2016).

The term Anthropocene prevailed, but whether the Anthropocene is an actual geological epoch, and when this new moment in Earth's history might have started are still contentious issues. The validity of a Global boundary stratotype section and point – GSSP – (or “golden spike” in lay terms) that demarcates the Anthropocene has been disputed (Gibbard et al., 2022), and some consider the Anthropocene to be an event, not an epoch (Merritts et al., 2023). Yet, the Anthropocene Working Group (AWG) from the Subcommission on Quaternary Stratigraphy (SQS) of the International Commission on Stratigraphy (ICS) has gathered extensive evidence for a GSSP dated from the mid-20th century (Head et al., 2022). Surprisingly, the Crawford Lake (situated just 136 Km from London, Ontario) has been proposed by the AWG as an official GSSP candidate site for the beginning of the Anthropocene (https://shorturl.at/bmxMZ).

Despite ongoing terminological disagreements around the Anthropocene, one fact remains undisputed – the species *Homo sapiens* has changed the planet in unprecedented ways. From ancient megafauna extinctions to the present-day obliteration of global resources, the socioeconomic and ecological meaning(s) of the term Anthropocene could not be clearer. Modern life as we know it, which started with the Great Acceleration in post-World War II (Steffen et al., 2007) has created a collective cognitive dissonance – the erroneous idea that we are not only detached from the fabric of life, but also Earth is ours to take. Ironically, as we clutter the planet with undegradable and toxic pollutants, we are creating the clearest GSSP of our time - a global stratum of technofossils (Zalasiewicz et al., 2014).

Students and young scientists are entering a world facing growing crises and a scientific community struggling to find solutions and inform the public or policymakers. A major goal of OE3C 2023 was to bring agency to those in the field that may feel the most powerless, as they have the most to lose while preparing for a lifetime amidst planetary crises.

## Engagement and imagery

In addition to talks, posters, and the panel discussion, OE3C 2023 featured a variety of visual materials, using artistic expression, anthropomorphism, humour, and narrative to bring lightheartedness and insight to the dark topic of the Anthropocene. Images can be visually appealing but also full of meaning (Smith, 1988; Chandler, 2007), while delivering educative information (Mayer and Gallini, 1990; Kools et al., 2006). Comics and humour are useful ways to increase enjoyment and retention of details (Yeo et al., 2020), especially because of the role of narrative and investment in following characters through their story (Dahlstrom, 2014; Farinella, 2018a,b). Cartoons and characters can use signs and expressions from humans as anthropomorphism to convey relatable human information (Lorimer, 2007; Wang et al., 2020) and increase relationship and interaction with the topic (Airenti, 2018; Conrad et al., 2020). This anthropomorphic interaction may be especially useful for exploring topics more distant from the human experience (Chan, 2012; Bruni et al., 2018).

**Fig. 2.**
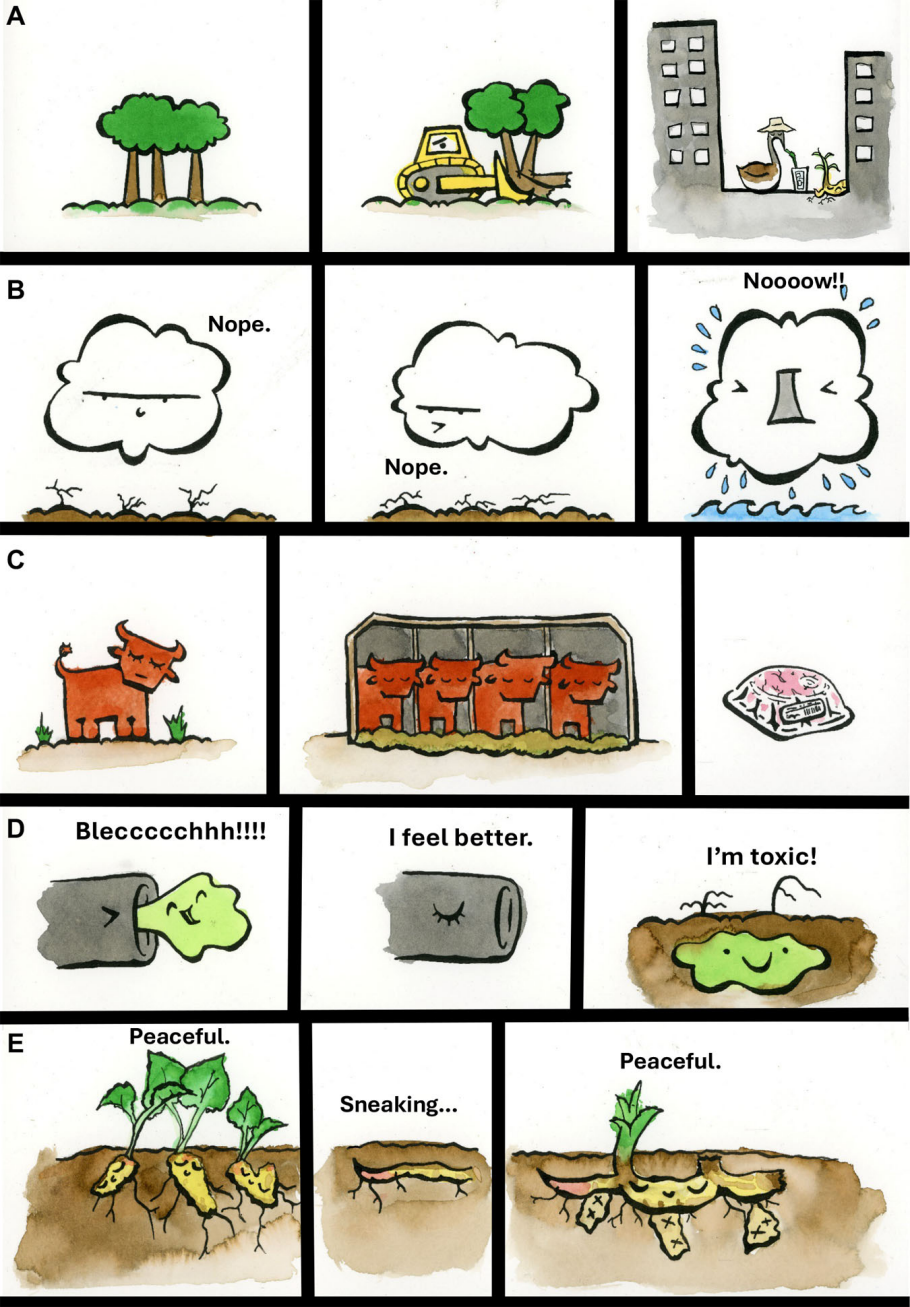
**Cute and emotive comic strips showcasing common scenarios typical of the Anthropocene.** (A) Illustration of land-use change, deforestation, and urbanization. (B) Impression of hydrological intensification, drought, and flooding. (C) Representation of industrial farming. (D) Depiction of toxic wastewater polluting soil. (E) Portrait of underground invasive species in action. Throughout the conference (including promotional materials - https://oe3c2023.com/), different snippets of the OE3C 2023 artwork were used to convey powerful and relatable messages pertaining to the Anthropocene. More information about the artist (Curtis Lubbe) can be found at: https://ecoevocommunity.nature.com/users/curtis-lubbe.

We used colourful characters depicting native flora and fauna as well as more complex topics such as land-use-change, hydrological intensification, plastics, and even scientific discovery itself as a lab coat-clad flask of mystery fluid. These characters featured a range of expressions with varying degrees of grace and humour when facing anthropogenic climate change, such as a groundhog sledding down an eroded hill or a goose enjoying a cool beverage in an urban heat island (https://oe3c2023.com/). We need every tool in our arsenal to inform and educate, and this can include the element of fun as we illustrate and tell our stories through the Anthropocene. Fig. 2 represents a sample of the storylines that guided the creation of the colloquium artwork.

## Panel discussion

Extreme weather events continue to increase in frequency (Najibi and Devineni, 2018), and our planetary crises will necessitate a complex range of actions for mitigation (Abbass et al., 2022; Parmesan et al., 2022). Although an international plan to combat climate change was adopted in 2015 (Kickbusch and Alakija, 2023), if we continue business as usual, not a single UN Sustainable Development Goal (SDG) will have been achieved by 2030 (Independent Group of Scientists, 2023). SDG 13, 14, 15 (Climate Action, Life Below Water, and Life on Land, respectively) are all in a path of deterioration or limited to no progress (Malekpour et al., 2023). On top of that, online platforms teeming with live global environmental data, such as Our World in Data (https://ourworldindata.org/) and Climate Watch (https://www.climatewatchdata.org/), only exacerbate the feelings of overwhelm and powerlessness. Therefore, the OE3C 2023 featured a panel discussion, presenting a big-picture view of the current climate crisis. The main topics discussed during the panel are distilled in a glossary below. The entire panel, with discussions that covered these topics and related concepts, can be found on the Western Evolution seminar series YouTube channel (part I – https://youtu.be/ocJ2dp8PfFo?si=oAhcjKJClo9-YLQs; part II – https://youtu.be/3rW0RVnsxwQ?si=CxqCwJtWMR30VFdh). Panelists' remarks are summarized (non-verbatim) after the glossary.

### Planetary boundaries

Nine planetary boundaries for the safe existence of our species on Earth were first proposed in 2009 (Rockström et al., 2009): climate change, novel entities, stratospheric ozone depletion, atmospheric aerosol loading, ocean acidification, biogeochemical flows, freshwater use, land-system change, and biosphere integrity. At the time of their debut, three of the nine boundaries had been crossed. By 2015, four boundaries had been surpassed (Steffen et al., 2015), and as of writing, six boundaries have been breached (Richardson et al., 2023). Boundaries have not been mildly or moderately crossed but have been transgressed mostly into high-risk zones. Although all boundaries are connected and can have spill-over effects by reaching tipping points (see below), the biosphere integrity boundary should serve as red alert signal to biologists. This boundary was already in the first three boundaries that had been crossed in the first estimate, and things have only declined precipitously since then.

### Tipping points

Tipping points can be broadly defined as a “point of no return” in a complex system. Climate tipping points were formally proposed in 2008 (Lenton et al., 2008), and several studies around the tipping point concept have been published since then. Whether or not the concept can be adequately applied to biodiversity at a global scale has been discussed many times (Lenton and Williams, 2013), but the capacity of climate tipping points (CTPs) to “turn the planetary tables” irreversibly is beyond dispute (Lenton et al., 2019). Importantly, CTPs are thresholds pertaining to Climate Tipping Elements, which can be divided in Global Core and Regional Impact tipping elements (Armstrong McKay et al., 2022). Perhaps one of the most discussed tipping elements is the Atlantic meridional overturning circulation (AMOC) and we may reach its tipping point already by the middle of the 21st century (Ditlevsen and Ditlevsen, 2023).

### Living planet index (LPI)

The loss of biodiversity caused by anthropogenic factors has been documented extensively both in the general media (https://www.theguardian.com/environment/2022/dec/06/the-biodiversity-crisis-in-numbers-a-visual-guide-aoe) and in the scientific literature (Hautier et al., 2015). The main contributor to the current biodiversity crisis is land use change (Newbold et al., 2016), but the impacts of other human activities on biodiversity (loss) are pervasive and plentiful (Otto, 2018). Anthropocene defaunation, has been comprehensively documented (Dirzo et al., 2014). The more we destroy Earth's habitats, the greater is our extinction debt such as, the uncounted, yet expected, future loss of species because of present-day habitat loss and deterioration (Tilman et al., 1994). The need and applicability of biodiversity indices are not new, but the living planet index (LPI; https://livingplanetindex.org/) has been particularly alarming since the publication of the Living Planet Report in 2022 (https://livingplanet.panda.org/en-US/). The report shows a staggering 69% overall decline in biodiversity between 1970 and 2018 (Ledger et al., 2023). It is noteworthy that the LPI assessed only major vertebrate lineages. Biologists and policy makers should not forget that we live in a microbial world, and efforts to study the climate impacts on microbial biodiversity should not be overlooked (Jansson and Wu, 2023).

### Carbon bombs

After decades of cynical propaganda, pervasive lobbying, and unabated carbon emissions, the world's major fossil fuel companies continue to contribute overwhelmingly to atmospheric CO_2_ increases. Investigations by The Guardian (https://www.theguardian.com/environment/ng-interactive/2022/may/11/fossil-fuel-carbon-bombs-climate-breakdown-oil-gas) and academic studies (Kühne et al., 2022) unravelled upcoming fossil fuel extraction projects (the so-called “carbon bombs”) that have a carbon footprint of at least 1 billion tonnes of CO_2_ each. In total, 195 carbon bombs have been found amongst the major fossil fuel companies (e.g. ExxonMobil, Shell, Petrobras, etc) across the globe. Sixty percent of these carbon bombs have already been “detonated”, meaning that their extraction projects are currently in operation. If all these bombs go unchecked and are set off, the Paris Agreement goal to keep global warming under 1.5°C is certain not to be reached.

### Geoengineering and promising technologies

Amidst such a bleak outlook, it is all too easy to hope that technology will save us. Paul Crutzen, the avid proponent of the term Anthropocene, defended the need of geoengineering technologies to save planet Earth from the Anthropocene assaults (Crutzen, 2006b). Broadly speaking, geoengineering (or climate geoengineering) is an umbrella term that encompasses several proposed technologies that could avert global warming without changing our carbon emissions (Vaughan and Lenton, 2011). The proposed geoengineering technologies can be divided in two types: carbon dioxide removal (CDR) or solar radiation management (SRM) (Lawrence et al., 2018). Although some CDR technologies are already implemented by the private sector, geoengineering is incipient and highly risky (Schäfer and Low, 2014). Given the possible global-wide adverse effects of geoengineering, the Climate Overshoot Commission has just called for a moratorium on SRM technologies (https://www.overshootcommission.org/report). In fact, it is not too difficult to imagine what could happen if plutocrats decided to use their power to apply SRM and CDR technologies at their will (Greta, 2023). A case in point is Russ George and his unscientific iron fertilization attempts (Tollefson, 2017).

### Panellists' remarks

The framework of these benchmarks, and the examination of extreme circumstances (as well as the use of extreme conditions in planetary research) can be difficult for scientists to investigate. We need to be mindful of extremes and the use of our measures and treatments, so that they accurately assess our changing climate. The quest for greater impact of publications can cause a struggle when facing the complex and sometimes subtle results of experiments examining climate change. Even though the impacts of climate change may be complex and even subtle on a small scale, as these conditions occur globally, they can scale-up to cause major impacts. Panellists highlighted that we must examine the response curve and not just control versus treatment. We should even be mindful of what we can call a control, as we investigate systems that have already begun changing. In reality, we struggle to document and understand the range of species and the state of ecosystems that existed before global warming. Although this may be seen as observational and less novel or innovative, this is vital knowledge that will be needed to mitigate climate change. Importantly, this information includes understanding carbon sequestration potential.

The Anthropocene as a concept can be difficult for scientists, especially as we place ourselves and our work inside the larger fight against anthropogenic forces, as mentioned by all of the panellists. Those taking practical action to study and help mitigate the effects of anthropogenic climate change can have different focuses, and the definition of the Anthropocene as a term is complicated by the terminology of the work of biologists. Trying to assess what to use as ‘ambient conditions’ during the changes to our natural settings is a major challenge as well. All panellists also highlighted the interconnectedness of our communities and work. We need a variety of approaches, especially examining and integrating as much understanding from the field as possible. In the future, scientists need to be more integrated in their approaches, to keep these key sensitivities of the planet in mind and to move forward in crossing them off our list of concerns. All panellists also highlighted the need for engagement in educating people and driving policy, as well as a concern for the communities, both locally and globally, that will be most affected by climate change. The final tone of the discussion was hopeful, as the panellists considered the promise they see in their continued work to understand and mitigate the effects of climate change. The enthusiasm and dedication of young colleagues and students, such as those asking questions during the panel, was also highlighted.

## Emerging themes and state of the field: future steps for ecology, ethology, and evolution

By the end of the OE3C 2023, the overall realization was that ecology, ethology, and evolution are unique disciplines, but they all share the same study subject – life. Hence, these disciplines can be amalgamated under the term Anthropocene. Because the Anthropocene brings ever-increasing challenges to the existence of life, biologists find themselves in an unnerving yet formidable position to effect positive change. Some of these challenges and potential positive changes are highlighted below.

### Ecology

Ecology in the Anthropocene must embrace multifactorial ecological experiments (both in the laboratory and in the field). As several planetary boundaries have been crossed, and global and regional tipping points are looming ever closer, all forms of life (and viruses) have and will continue to experience multifaceted stressors. We must carefully assess past, current, and potential future conditions to gain as much information while we still can. Ecologists should also aim to tease apart not only what types of stressors will be at play, but where, when, and how these stressors interact with one another (Simmons et al., 2021).

### Ethology

Ethological studies should continue to explore how animals change their behaviour (both locally and temporally) in response to climate change, habitat fragmentation, noise pollution, and so on. Anecdotally, we observed that most of the young colloquium participants were unfamiliar with the term Ethology. This is concerning and needs remedy. As discipline-specific vocabulary can impede effective communication, ethologists should perhaps consider collectively what term(s) should be used to best describe their field of study (Bueno-Guerra, 2021).

### Evolution

The biggest challenge for evolutionary studies in the Anthropocene will be to tease apart plasticity from evolutionary change (Hendry et al., 2017). More examples of how anthropogenic factors impact (i.e. speed up or slow down) the evolution of species are expected. After all, humans are now considered the “world's greatest evolutionary force” (Palumbi, 2001), and a dangerous one at that. We have, for instance, driven the evolution of resistance to antibiotics, pesticides, and other drugs.

From the discussions that happened during the conference, it became clear that the 3 E's are expected to work even closer with one another during the Anthropocene. Although, the situation is dire, none of our knowledge can be put to good use, if we do not communicate it widely and loudly. Scientists, biologists chiefly amongst them, should work on the promotion of their findings. If knowledge is power, and with power comes responsibility, we have the moral obligation to inform the public and strive for change. In the end, biologists shall not forget that their profession and passion rely on the existence of life on Earth. If we want to continue to contemplate the wonders of the natural world, we must first defend them.

## Diversity and inclusion statement

We are fully aware of the (implicit and explicit) systemic barriers that prevent the equitable development of all members of our society. Therefore, the Organizing Committee wants to make a clear statement on Equity, Diversity, and Inclusion. We welcome students and professionals from all walks of life. We know we cannot change the world in one conference, but we can take the necessary steps to promote the change that is needed. Factors such as age, nationality, ethnicity, religion, gender identity, sex, sexual orientation, socioeconomic status, physical and neurological differences, marital status, parental status, and others all contribute to privileges and/or prejudices. These privileges and prejudices often determine who has the power to make decisions. Accordingly, representativeness must be tackled and embraced fully. We hope that the environment and the discussions fostered during the conference created not only a welcoming and safe space for all, but also imparted change in the way participants think about our (collective and individual) positionalities.
